# Human Epidermal Growth Factor Receptor-2 Gastric Adenocarcinoma: Expanding Therapy of a Recognized Target

**DOI:** 10.3390/cancers15215180

**Published:** 2023-10-27

**Authors:** Jane E. Rogers, Kohei Yamashita, Matheus Sewastjanow-Silva, Allison Trail, Rebecca E. Waters, Jaffer Ajani

**Affiliations:** 1 Pharmacy Clinical Programs, U.T. M.D. Anderson Cancer Center, Houston, TX 77030, USA; 2 Department of Gastrointestinal Medical Oncology, U.T. M.D. Anderson Cancer Center, Houston, TX 77030, USA; kyamashita@mdanderson.org (K.Y.); msewastjanow@mdanderson.org (M.S.-S.); atrail@mdanderson.org (A.T.); 3 Department of Pathology, U.T. M.D. Anderson Cancer Center, Houston, TX 77030, USA; rwaters@mdanderson.org

**Keywords:** gastrointestinal neoplasms, human epidermal growth factor receptor-2, trastuzumab, trastuzumab deruxtecan, ZW25, margetuximab

## Abstract

**Simple Summary:**

Human epidermal growth factor receptor-2 (HER2) is a well-known target for approximately 15% of gastric adenocarcinomas (GACs). Although a plethora of HER2-targeted agents are marketed, currently only two agents are approved for GAC. These two agents are used only in the metastatic setting. Trastuzumab is utilized in combination with front-line chemotherapy, and trastuzumab deruxtecan is given following failure of trastuzumab therapy. Questions remain as to why HER2 biology is different in different tumor types. Here, we discuss past HER2-targeted failures, resistance patterns, and new agents on the investigative horizon.

**Abstract:**

Human epidermal growth factor receptor-2 (HER2) is a well-known cancer target. Many HER2-targeted agents are marketed and being investigated. Unfortunately, these therapies lack consistent responses and outcomes amongst different tumors. Questions remain as to why HER2 biology is different in different tumor types. Gastric adenocarcinomas (GACs) demonstrate both intra- and inter-tumor HER2 expression heterogeneity and show discordance amongst primary and metastatic disease sites. This creates barriers in determining HER2 agents’ effectiveness and contributes to the failure of some HER2-targeted agents in the treatment of HER2-positive advanced GACs. Trastuzumab deruxtecan, an antibody drug conjugate of trastuzumab with a topoisomerase inhibitor, was recently approved for the treatment of refractory HER2-positive advanced GAC patients. There are exciting and newer therapies under investigation. Examining resistance patterns (both adaptive and acquired) along with establishing a better understanding of the intra- and inter-tumor heterogeneity is necessary to ensure successful progress. Here we review the current status of HER2-targeted therapy in GACs. We additionally review newer therapies under investigation and their potential role in HER2 GACs.

## 1. Introduction

Metastatic gastric adenocarcinomas (GACs) carry a poor prognosis with limited therapy options after first-line failure [1]. Some GACs (~15%) over-express human epidermal growth factor receptor-2 (HER2), making them candidates for HER2-targeted therapy. According to the guidelines, HER2 over-expressing GACs are classified by HER2 protein 3+ via immunohistochemistry (IHC), HER2 protein 2+ via IHC+ with an ERBB2/CEP17 ratio ≥ 2 using fluorescence in situ hybridization (FISH), or an average of *ERBB2* copy number ≥ 6 signals/cell. Newer HER2 therapies are challenging these designations by exploring effectiveness in those with lower expression (or assessments using other platforms such as liquid biopsy or Next Gen Sequencing). Our review focuses on the current understanding of HER2 agents in HER2-positive advanced GAC management. We review historical setbacks in HER2 GAC along with current barriers in HER2 pathology testing. Additionally, we discuss newer agents and resistance patterns. 

## 2. Historical and Current HER2-Positive Advanced GAC Management

It is unclear if HER2 is a prognostic factor in GACs; however, it is a clear target for HER2-directed therapy [1]. Trastuzumab is a humanized monoclonal antibody (mAb) that binds to the extracellular domain (domain IV) of HER2. It has been incorporated into the treatment of metastatic HER2 GAC/gastroesophageal junction cancer (GEJ) since results of the 2010 ToGA trial showed a modest overall survival (OS) benefit. ToGA was a multicenter phase 3 randomized trial evaluating front-line fluoropyrimidine + cisplatin +/− trastuzumab in HER2-positive advanced GACs [2]. HER2 positivity was per IHC 3+ or the FISH HER2:CEP17 ratio ≥ 2. The trastuzumab plus chemotherapy arm (n = 294) had a reported median OS of 13.8 months (95% CI 12–16 months) vs. 11.1 months (95% CI 10–13 months) in the chemotherapy-alone arm (n = 290, hazard ratio (HR) 0.74; 95% CI 0.60–0.91; *p* = 0.0046). Median progression-free survival (PFS) was improved (6.7 months in the trastuzumab plus chemotherapy arm vs. 5.5. months in the chemotherapy-alone arm, HR of 0.71, 95% CI of 0.59–0.85; *p* = 0.0002). The overall response rate (ORR) was also higher (47% in the trastuzumab plus chemotherapy arm vs. 35% in the chemotherapy-alone arm; *p* = 0.0017). This OS improvement led to FDA approval and the first biologic agent approved for use in GACs. Of note, those (n = 131) who were FISH positive but had IHC 0/1+ disease did not have an improved OS with the addition of trastuzumab (10 months vs. 8.7 months, HR 1.07). This group of patients represented ~20–25% of each group.

Unfortunately, for over a decade after trastuzumab, HER2-targeted agents alone and in combinations failed against advanced HER2-positive GAC. The JACOB (pertuzumab plus trastuzumab), LOGiC (lapatinib plus chemotherapy), TyTAN (lapatinib), and GATSBY (trastuzumab emtansine) trials all failed to meet their primary endpoint [3,4,5,6,7]. These trials are summarized in Table 1. TyTAN explored lapatinib, an oral tyrosine kinase that targets domains of epidermal growth factor (EGFR1) and HER2, in combination with paclitaxel in the second-line metastatic setting in an Asian GAC population [5]. The majority were untreated with trastuzumab (6% previously treated with trastuzumab). The authors included patients based on HER2 positivity where FISH HER2:CEP17 ratio ≥ 2. Approximately 35% in each group were FISH positive but had IHC 0/1+ disease. Median OS was not improved in the overall population (11 months vs. 8.9 months, *p* = 0.1044), which was the primary endpoint. Median PFS was also not improved statistically (5.5 months vs. 4.4 months, *p* = 0.2441). A statistical difference was seen in the Chinese population through a subgroup analysis. A review of the subgroup analysis also showed those who had IHC 0/1+ might have impacted results as those with IHC 3+ and were FISH positive showed more meaningful clinical prolongation of OS. LOGiC, a phase 3 multicenter, international trial, investigated capecitabine + oxaliplatin + lapatinib (n = 249) or placebo (n = 238) in the front-line metastatic setting for HER2-positive GAC, GEJ, or esophageal adenocarcinoma (EAC) patients [4]. FISH showing a ≥2 ratio was considered positive, although 20% in the lapatinib arm and 14% in the placebo arm had IHC 0 or 1+, respectively. Median OS, which was the primary endpoint, was not statistically improved (12.2 months vs. 10.5 months, *p* = 0.3492). Median PFS (6 months vs. 5.4 months, *p* = 0.0381) and response rate (53% vs. 39%, *p* = 0.0031) reached statistical significance. In the subgroup analysis, Asian patients showed more benefit. The authors reported no OS correlation based on IHC score. The JACOB trial was a phase 3 multicenter, international, double-blind, randomized, placebo-controlled trial evaluating pertuzumab, a monoclonal antibody directed at the extracellular dimerization domain (domain II) of HER2 [3]. HER2-positive GAC or GEJ patients (n = 780) were randomized to pertuzumab plus trastuzumab with chemotherapy (n = 388) or placebo plus trastuzumab with chemotherapy (n = 392). HER2 positive was defined as IHC 3+ or 2+ with ISH positive. Median OS, which was the study’s primary endpoint, was not statistically significant (18.1 months vs. 14.2 months, HR 0.85, 95% CI 0.72–0.99). Median PFS was improved (8.5 months vs. 7.2 months, HR 0.73: 95% CI 0.62–0.85), and objective response was larger (56.7% vs. 48.6%, *p* = 0.026). OS was longer for patients with homogenous HER2 IHC staining patterns (n = 279 in the IHC3+ subgroup) vs. heterogeneous (n = 125 in the IHC3+ subgroup) or focal staining (n = 117 in the IHC3+ subgroup). GATSBY, a phase 2/3 international trial, evaluated trastuzumab emtansine, an antibody drug conjugate of trastuzumab attached to the DM1 microtubule linker (n = 228) vs. taxane therapy (n = 117) in the second-line HER2 GAC or GEJ metastatic setting [6]. Most patients (~80% in each group) had received prior HER2 therapy and the majority had IHC 2+ or 3+/ISH positive disease (~90% in each group). Median OS, which was the primary endpoint, was not improved (7.9 months vs. 8.6 months, *p* = 0.86). Median PFS was also not improved (2.7 months vs. 2.9 months, *p* = 0.31). A biomarker subgroup analysis performed by Shah et al. showed median OS was longer in those with HER IHC3+ (9.5 months vs. 8.3 months, HR 0.99: 95% CI 0.68–1.43) than those with IHC 2+ (5.2 months vs. 9.2 months, HR 1.53: 95% CI 0.94–2.50) [7]. Those with homogeneous staining showed a trend toward increased OS. However, trastuzumab emtansine was not associated with superior OS vs. taxane in any subgroup analysis. The lessons learned by looking back at these failed studies are the importance of patient selection for trial enrolment and focusing on IHC 3+ or IHC 2+/FISH + patients. Additionally, these articles point out the heterogenous nature of GACs, both via interpatient variability (geographic location) and intra-patient variability (HER2 heterogeneous staining positivity).

After trastuzumab’s approval, additional HER2-targeted approvals were stagnant until 2021 with DESTINY-Gastric01. DESTINY-Gastric01, a phase 2 trial, led to the FDA approval of trastuzumab deruxtecan, an antibody drug conjugate (ADC) of trastuzumab with a topoisomerase inhibitor [8,9]. Trastuzumab deruxtecan was approved for advanced HER2-positive (IHC3+ or IHC2+/ISH+) GACs in the second or later lines (after trastuzumab failure) in an Asian population (79.7% were Japanese and 20.3% were Korean) [8]. Trastuzumab deruxtecan (n = 125) was compared to physician choice chemotherapy (irinotecan or paclitaxel) (n = 62). Outcomes were improved with trastuzumab deruxtecan (median OS 12.5 vs. 8.9 months; HR = 0.60 (95% CI, 0.42–0.86); 12-month OS was 52.2% vs. 29.7%; ORR was 51.3% vs. 14.3%, *p* < 0.0001; median PFS 5.6 months vs. 3.5 months HR = 0.47 (95% CI 0.31–0.71)). Low HER2-positive (IHC 2+/ISH− or IHC 1+) patients were included in a separate exploratory analysis [10]. The exploratory analysis of those with low HER2 expression (cohort 1IHC 2+/ISH negative = 21; cohort 2 IHC1+ = 24) showed the ORR was 26.3% and 9.5% in cohorts 1 and 2, respectively. Reduced tumor size was seen in both cohorts at ~60%. Median OS was 7.8 months in cohort 1 and 8.5 months in cohort 2, and median PFS was 4.4 months in cohort 1 and 2.8 months in cohort 2. The DESTINY-Gastric02 trial was a phase 2 trial of trastuzumab deruxtecan in the USA and Europe HER2-positive (IHC 3+ or IHC 2+ with ISH +) GAC or GEJ population (n = 79) [11]. The ORR was 30%, median PFS was 5.6 months, and median OS was 12.1 months. Trastuzumab deruxtecan is currently under investigation in DESTINY-Gastric04, a phase 3 trial to confirm results [12]. Trastuzumab deruxtecan shows the potential of HER2 ADCs in HER2-positive GAC; however, this drug can be very toxic and careful patient selection, education, and monitoring is recommended. As evident in breast cancer, low HER2 positivity might also be a potential responder to this agent and needs further exploration [13].

Recently, KEYNOTE-811, a multicenter phase 3 randomized trial, changed front-line therapy for advanced HER2-positive (IHC 3+ or IHC 2+/ISH+) GAC patients in the USA [14]. KEYNOTE-811 evaluated front-line standard of care (SOC) trastuzumab + fluoropyrimidine + platinum (cisplatin or oxaliplatin) +/− pembrolizumab. Programmed death ligand-1 (PD-L1) expression was not an inclusion criterion. The pembrolizumab plus SOC arm (n = 133) had an overall response rate (ORR) of 74.4% (95% CI 66.2–81.6%) compared to the 51.9% ORR (95% CI 43–60.7%) in the placebo plus SOC arm (n = 131), in the interim response evaluation of limited number of patients. Complete responses were observed in 11.3% of the pembrolizumab plus SOC arm compared to 3.1% in the placebo plus SOC arm. The duration of response was slightly longer in the pembrolizumab arm. We look forward to the full data from this study.

Currently, standard of care for HER2-positive advanced GACs is provided with front-line trastuzumab + pembrolizumab + fluoropyrimidine + platinum followed by trastuzumab deruxtecan after progression [1]. Results from all these evaluations show that there are patients who will lack a response (~50% ORR with trastuzumab plus chemotherapy; ~40% with trastuzumab deruxtecan) to these HER2-targeted agents, showing intrinsic resistance [2,8]. Additionally, most patients will eventually acquire resistance and have progression of cancer on these therapies. To advance this area, examination of resistance patterns is needed.

## 3. GAC HER2 Resistance Patterns: Tumor HER2 Heterogeneity and HER2 Protein Expression Loss

HER2 expression disparities are present in GACs, as reported in upwards of ~80% of cases, which is well over the proportion seen in breast cancer [15]. Zhang et al. reviewed GAC patients (n = 618) for intra-tumor and inter-tumor heterogeneity [16]. Two formalin-fixed paraffin-embedded (FFPE) tumor-containing blocks per patient were reviewed for HER2 IHC staining and correlated to clinicopathological features. The authors showed that dual block assays increased HER2 IHC 3+ and 2+ compared to a single FFPE block. Single tissue section showed that ~50% of cases had intra-tumor HER2 heterogeneity and 30% showed inter-tumoral heterogeneity between the patient’s two blocks. Kaito et al. reported on the clinical significance of intra-tumor heterogeneity in GAC patients receiving trastuzumab-containing therapy [17]. Patients with every biopsy portion having HER2 positivity via IHC were defined as the HOMO group and those that had any portion HER2 negative were the HETERO group. The HOMO group had significantly better outcomes (ORR 79.5% vs. 35.7%; median PFS 7.9 months vs. 2.5 months; median OS 25.7 months vs. 12.5 months). To account for intra-tumor heterogeneity, it is recommended to perform a minimum of five biopsies [15]. A key for the trial development might be to separate these groups based on expression to understand agent effectiveness in certain subgroups, but this can be very challenging. Additionally, HER2 expression discrepancies can exist between primary tumor and metastatic sites. Discordance has been seen in breast cancer and appears to be variable in GAC [18]. Peng et al. conducted a meta-analysis of discordance reported in primary GAC and metastatic disease. The authors found 2–24% discordance rates. This discordance leads to further challenges in the exploration of these agents as it is often not common practice to take multiple tissue biopsies from the primary and metastatic sites. These limitations in current GAC HER2 analysis were also discussed by Leni et al. [19]. The authors propose a role for circulating tumor DNA (ctDNA), which may help with some of the limitations seen, early detection of acquired resistance, and monitoring of treatment response. We await more study with ctDNA in this area. An additional area of concern is that of variability amongst testing across pathology centers, as described by Baretton et al. The authors suggested HER2 testing quality should consider primary tumor location, testing method and rate, and tumor characteristics [20].

Another concept is that of a potential loss of HER2 expression by the tumor, as studies have shown a loss of HER2 in those progressing with therapy in both breast cancer and GAC. Pietrantonio et al. reported a 32% loss of HER2 post-trastuzumab [21]. Seo et al. reported that this concept can impact second-line anti-HER2 treatment [22]. The authors reported a 29.1% loss of HER2 positivity post-trastuzumab progression biopsy. Patients receiving second-line trastuzumab emtansine who had shown HER2 negative conversion (n = 3) showed no response to trastuzumab emtansine (0% ORR; short PFS 1.2–3.4 months). This leads to questions of whether checking tumor biopsy or liquid biopsy at time of progression might be a path forward to determine the correct patients selected for HER2 treatment.

Other resistance patterns include mutations in the receptor, which can lead to a lack of the target binding site [15,23]. Potential resistance has been seen due to impaired HER2 receptor binding though coverage of different proteins or a loss of the binding region. Alternate signaling pathways or activation of downstream signaling (EGFR, MAPK, MET upregulation, PI3K/AKT, PTEN loss, FGFR) have also been theorized as contributing to resistance. Collectively, along with the smaller population of HER2-positive GAC, all these factors are likely why many HER2 targets failed in GACs compared to those of other HER2-positive solid tumors, particularly breast cancer.

## 4. Newer HER2 Therapies

### 4.1. Antibody Drug Conjugates (ADCs)

ADCs are a novel drug design in which antibodies are chemically linked to cytotoxic therapy [15,23,24,25]. The antibody component exerts its anti-tumor effects by recognizing the antigen on the target cells, facilitating the formation of an antigen–antibody conjugate, which allows the cytotoxic payload to be rapidly internalized, leading to release of the cytotoxic component [25]. Ideally, this mechanism should reduce off-target toxicity; however, as mentioned previously, trastuzumab deruxtecan, an ADC that consists of trastuzumab with a topoisomerase inhibitor, carries substantial toxicity [10,11]. The hope is that newer generations of ADCs and continued development in this area will yield safer agents. Additional HER2 ADCs are being explored in HER2-positive GAC. RC48 is an ADC composed of hertuzumab, an anti-HER2 mAb conjugated to a microtubule inhibitor, monomethylauristatin E (MMAE) [26,27]. A phase 2 trial in ICH 2+/3+ advanced GAC patients in the refractory setting showed an ORR of 24.8%, median PFS of 4.1 months, and median OS of 7.9 months [25,26,27]. Those with HER2 IHC 2+/FISH− showed an ORR of 16.7%. Of note, RC48 was approved in China for GAC. Phase 3 in this population is under investigation using NCT04714190 [28]. Other HER2 ADCs are being explored in solid tumors. Preliminary results of ZW49 (auristatin) in heavily pretreated HER2-positive solid tumor patients showed an ORR of 31% with disease control of 72% [29,30]. For the GAC patients (n = 11), the ORR was 37% with a disease control rate of 73%. ARX788 (amberstatin conjugate) showed encouraging phase 1 results in HER2 refractory GAC patients (n = 30) with an ORR of 37.9%, disease control of 55.2%, median PFS of 4.1 months, and median OS of 10.7 months [31]. ARX788 was granted orphan drug status with the FDA in 2021 [32]. Examples along with their cytotoxic payload include MRG002 (microtubule disrupting agent monomethyl auristatin E), SYD985 (duocarmyicin), PF-06804103 (Aur0101), FS-1502 (monomethyl auristatin F), GQ1001 (DM1), A166 (microtubule cytotoxic agent), XMT-1522 (auristatin), BDC-1001 (toll-like receptor), ALT-P7 (monomethyl auristatin E), and SBT6050 (toll-like receptor) [15,24,25]. Trial examples of these agents are described in Table 2 [28,33,34,35,36,37,38,39,40,41].

### 4.2. Antibodies

Zanidatamab (ZW25), a HER2-targeted bispecific antibody, has emerged as a promising therapy. It binds to two extracellular domains of HER2, extracellular domain IV and II. These are the same domains targeted by trastuzumab and pertuzumab, respectively. Meric-Bernstam et al. published phase 1 results in HER2 IHC 3+ or 2+ advanced refractory (median prior therapies = 2–3) GACs [42]. Parts 1 and 2 were given single-agent zanidatamab (n = 36), whereas part 3 (n = 26) utilized zanidatamab in combination with a fluoropyrimidine or a taxane. Most patients had prior HER2 therapies (>90%). The ORR was 38% for the single-agent parts and 60% for zanidatamab + chemotherapy, with a median duration of response of 6 months (95% CI 1.9–9.2 months) and 8.9 months (95% CI 3.5-NE months), respectively. Ku et al. reported preliminary results on phase 2 of zanidatamab in combination with front-line fluoropyrimidine plus platinum HER2 IHC 3+ or IHC 2+/FISH + advanced GAC [43]. For 28 patients, the outcomes showed a benefit (75% ORR, median duration of response of 16.4 months, and median PFS of 12 months). Further evaluations are underway with NCT03929666, a phase 2 trial with zanidatamab + chemotherapy, and HERIZON-GEA-01, a phase 3 trial with zanidatamab + chemotherapy +/− tislelizumab, an anti-PD-I antibody [44,45]. Of note, preliminary results of NCT03929666 have shown remarkable outcomes thus far. Zanidatamab + chemotherapy (CapeOx, FOLFOX, 5-FU + cisplatin) (n = 38) achieved an ORR of 79%, and 13% had stable disease, showing a disease control rate of 92% with a median duration of response of 20.4 months [46]. The median PFS was 12.5 months and median OS was NE. The 12-month OS was 88% and 18-month OS was 84%. We look forward to results of phase 3 HERIZON-GEA-01 [45].

Margetuximab, a HER2 mAb similar to trastuzumab, has been engineered to increase affinity for the stimulatory Fc receptor (CD16A) and decrease affinity for the inhibitory Fc receptor (CD32B) on natural killer cells to increase antibody-dependent cellular cytotoxic response [47,48]. In vitro, margetuximab enhances the PD-1/PD-L1 axis expression and LAG-3 on natural killer and NK T cells. Blocking PD-1 would, in theory, enhance margetuximab NK cell activation, proliferation, and cytotoxicity. CP-MGAH22-05, a multicenter phase 1b/2 trial, combined margetuximab with pembrolizumab in refractory (1–2 previous therapies) HER2 IHC 3+ or IHC 2+/FISH+ advanced GAC (n = 95) [47]. PD-L1 expression was not an inclusion criterion but was explored (expression considered CPS ≥ 1). Overall outcomes were 18% ORR, median PFS of 2.73 months, and median OS of 12.48 months. HER2 amplification by ctDNA was associated with better ORR (HER2 amplification 31% ORR vs. HER2 amplification negative 6% ORR). Those with both HER2 IHC 3+ and PD-L1 expression also showed an ORR of 44%, and those with HER2 amplification by ctDNA with PD-L1 expression showed an ORR of 50%. The role of ctDNA in outcomes poses a unique question, namely whether following ctDNA expression during HER2 therapy may hold keys to establishing patients who can truly benefit. Interim analysis (n = 43) of the MAHOGANY trial, a phase 2/3 trial, was reported on the combination of margetuximab + retifanlimab, anti-PD-1 mAb, given as a front-line treatment for HER2 IHC 3+ and PD-L1 positive patients [49]. The best ORR was 52.5% with a median PFS of 6.4 months. MAHOGANY is also exploring the combination of margetuximab +/− tebotelimab, anti-LAG-3, and anti-PD-1 mAb [48].

KN026, a bispecific HER2 mAb targeting extracellular domains IV and II, has shown activity. Xu et al. showed phase 2 activity in two cohorts (cohort 1 (n = 25): IHC 3+ or IHC 2+/FISH +; cohort 2 (n = 14): IHC 0/1 +; FISH +) of refractory GAC and GEJ adenocarcinoma patients (≥one prior therapy) [50]. Cohort 1 reported an ORR of 55.6%, median PFS of 8.3 months, and median OS of 16.3 months. Of note, activity was seen in those that received prior HER2 therapy. Cohort 2 (n = 14) showed a minimal signal with 14% ORR, median PFS of 1.4 months, and median OS of 9.6 months, strengthening the importance of patient identification for trials. Dong et al. reported results of KN026 with KN046, anti-CTLA4/PD-L1 mAb in phase 1b GAC patients (n = 47). Eligibility included HER2 alterations (low expression, overexpression, mutation, or amplification), in naïve or refractory treatment. The ORR was 71.4% in the treatment naïve group and 37.5% in the refractory group. No response was seen in those with low HER2 expression or mutations. Of note, activity was still present in those that had prior HER2 and PD-1 agents. NCT05427383 is a current phase 2/3 trial evaluating KN046 in combination with chemotherapy (taxane or irinotecan) in the second-line HER2-positive advanced GAC [51].

### 4.3. Other Strategies

Promising results reported by ASPEN-01, a phase 1 trial, on ALX148, a CD47 inhibitor, show novel combination strategies [52]. ASPEN-01 studied the combination of ALX148 + trastuzumab (n = 20) or ALX148 + trastuzumab + ramucirumab + paclitaxel (n = 18) in the second-line setting for HER2-positive advanced GAC patients. Most patients had prior HER2-targeted agent exposure. The ORR was 72% in the arm with ramucirumab plus paclitaxel, and the ALX148 and trastuzumab arm ORR was 21%. ALX148 + trastuzumab + ramucirumab + paclitaxel is being evaluated in ASPEN-06, a phase 2/3 trial, in advanced HER2-positive GAC patients [53]. NCT05027139 is looking at ALX148 with ZW25 in HER2-positive solid tumor patients [54]. Triplet combinations with HER2 kinase inhibitors in combination with chemotherapy and PD-1 inhibition are also being explored, such as in NCT05111444 [55]. Additionally, studies of vaccine and chimeric antigen receptor T-cell (CART) HER2 targets are underway. Oral HER2 tyrosine kinase combination studies are also underway, including MOUNTAINEER-02, which is studying tucatinib in combination with trastuzumab, ramucirumab, and paclitaxel compared to ramucirumab and paclitaxel alone in the refractory setting [56].

Chen et al. recently published five potential hub genes that contribute to GAC [57]. The authors used gene expression profiles and an RNA-sequencing dataset of GAC acquired from the Gene Expression Omnibus dataset and The Cancer Genome Atlas dataset. Ten important gene sets associated with resistance were identified. Linking these discoveries with clinical practice is necessary for further success. We feel more understanding in this area will lead to advancements in HER2 resistance.

## 5. Conclusions

HER2 has been an oncologic target for decades. Despite historical advances in the knowledge of targeting HER2, there are still many unanswered questions surrounding HER2 in GAC patients. Overcoming patient selection issues for clinical trial development will help differentiate the effectiveness of agents in this population. Newer novel strategies are currently being explored. We are at an exciting time of discoveries in this subgroup of GAC patients. We recommend patients be enrolled in a HER2-targeted clinical trial when available.

## Figures and Tables

**Table 1:** Historical trials in metastatic HER2-positive gastric adenocarcinoma [2,3,4,5,6,7].

Trial Name	Study Population	Line of Therapy	Treatment	HER2 Expression	Outcomes
ToGA 2010	HER2 + advanced gastric and gastroesophageal junction adenocarcinoma	First Line	Trastuzumab + Fluoropyrimidine + Cisplatin (n = 294) Fluoropyrimidine + Cisplatin (n = 290)	Trastuzumab + Fluoropyrimidine + Cisplatin arm FISH positive/IHC 2+ or IHC 3+ (78%) Fluoropyrimidine + Cisplatin arm FISH positive/IHC 2+ or IHC 3 + (75%)	Median PFS 6.7 months vs. 5.5 months, *p* = 0.0004 Median OS 13.8 months vs. 11.1 months, *p* = 0.0046
TyTan 2014	HER2 + advanced gastric cancer	Second Line	Lapatinib + paclitaxel (n = 132) Paclitaxel (n = 129)	Lapatinib + Paclitaxel FISH positive IHC 2+ or IHC 3+ (64%) Paclitaxel FISH positive IHC 2+ or IHC 3+ (65%)	Median OS 11 months vs. 8.9 months, *p* = 0.1044 Median PFS 5.5 months vs. 4.4 months, *p* = 0.2441
JACOB 2018	HER2 + advanced gastric and gastroesophageal junction adenocarcinoma	First Line	Pertuzumab + Trastuzumab + Fluoropyrimidine + Cisplatin (n = 388) Trastuzumab + Fluoropyrimidine + Cisplatin (n = 392)	Pertuzumab + Trastuzumab + Fluoropyrimidine + Cisplatin ISH positive/IHC 2+ IHC3+ Trastuzumab + Fluoropyrimidine + Cisplatin ISH positive/IHC 2+ IHC3+	Median OS 18.1 months vs. 14.2 months, HR 0.85, 95% CI 0.72–0.99 Median PFS 8.5 months vs. 7.2 months, HR 0.73, 95% CI 0.62–0.85
LOGiC 2015	HER2+ advanced gastroesophageal adenocarcinomas	First Line	Lapatinib + Capecitabine + Oxaliplatin (n = 249) Capecitabine + Oxaliplatin (n = 238)	Lapatinib + Capecitabine + Oxaliplatin (n = 249) FISH positive/IHC 2+ (23%) FISH positive/IHC 3+ (57%) Capecitabine + Oxaliplatin (n = 238) FISH positive/IHC 2+ (21%) FISH positive/IHC 3+ (65%)	Median OS 12.2 months vs. 10.5 months, *p* = 0.3492 Median PFS 6 months vs. 5.4 months, *p* = 0.0381
GATSBY 2017	HER2+ advanced gastric and gastroesophageal junction adenocarcinoma	Second Line	Trastuzumab emtansine (n = 228) Taxane (docetaxel or paclitaxel) (n = 117)	FISH positive/IHC 2+ or IHC 3+	Median OS OS 7.9 months vs. 8.6 months, *p* = 0.86 Median PFS 2.9 vs. 2.7 months, *p* = 0.31

HER2: human epidermal growth factor receptor-2; IHC: immunohistochemistry; FISH: fluorescence in situ hybridization; PFS: progression-free survival; OS: overall survival.

**Table 2:** HER2-targeted antibody-drug conjugate examples currently under investigation [28,33,34,35,36,37,38,39,40,41].

Drug Name	HER2 bsAb	Trial Number	Phase	Population
RC48	Anti-HER2 + MMAE	NCT04714190 NCT05514158 NCT05982834	3 1 1/2	Locally advanced/metastatic HER2 GAC Locally advanced/metastatic HER2 GAC Metastatic HER2 GAC
ZW49	Anti-HER2 bsAb (ZW25) + Auristatin	NCT03821233	1	Advanced HER2-expressing cancers
MRG002	Anti-HER2 IgG1 + MMAE	NCT04492488 NCT05141747	1 2	Advanced HER2 solid tumors Locally advanced/metastatic HER2-positive/HER2 low GAC
FS-1502	Anti-HER2 + MMAF	NCT03944499	1	HER2-positive advanced breast or solid tumors
GQ1001	Anti-HER2 + DM1	NCT04450732	1	HER2-positive advanced solid tumors
ARX788	Modified Trastuzumab + MMAF	NCT03255070	1	HER2-positive advanced solid tumors
BDC-1001	Trastuzumab biosimilar + TLR7/8 agonist	NCT04278144	1/2	HER2-positive advanced solid tumors

bsAb (bispecific antibody); HER2: human epidermal growth factor receptor-2.
